# Pancreas divisum in a young patient with chronic abdominal pain as per radiological findings: A case report

**DOI:** 10.1002/ccr3.7798

**Published:** 2023-08-15

**Authors:** Prakash Kayastha, Biraj Pokhrel, Uma Bhatta, Roshan Pathak, Goody Jha, Sharma Paudel, Sundar Suwal, Dosti Regmi

**Affiliations:** ^1^ Department of Radiology Tribhuwan University Teaching Hospital Kathmandu Nepal; ^2^ Department of Pathology Kanti Children's Hospital Kathmandu Nepal; ^3^ Department of Radiology Kanti Children's Hospital Kathmandu Nepal

**Keywords:** chronic abdominal pain, crossing duct sign, pancreas divisum, pancreatitis

## Abstract

**Key Clinical Message:**

Pancreas divisum (PD) can be one of the causes of unexplained chronic abdominal pain. In PD, the dominant duct drains the majority of the pancreas via the minor papilla, which can be conveyed in the imaging as crossing duct sign.

**Abstract:**

We report a case a of 16‐year‐old man who presented with unexplained chronic abdominal pain. Contrast‐enhanced computed tomography and magnetic resonance cholangiopancreatography showed a bulky pancreas, but his pancreatic enzymes were normal. The crossing duct sign was prominent in imaging, which confirmed the diagnosis of PD.

## Graphical abstract

Contrast‐enhanced computed tomography, (coronal view) and magnetic resonance cholangiopancreatography (T2‐weighted) show the dominant dorsal duct crossing the intrapancreatic common bile duct giving crossing duct sign. This confirms the diagnosis of Pancreas divisum.

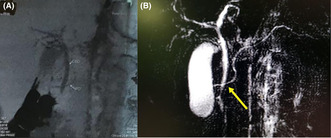

## 1 INTRODUCTION

Pancreas divisum (PD) is the most common ductal developmental anatomic variant of the pancreas. The incidence of PD in the population at autopsy series, endoscopic retrograde cholangiopancreatography (ERCP), and magnetic resonance cholangiopancreatography (MRCP) is 4%–14%, 3%–8%, and 9%, respectively.^1^, ^2^, ^3^, ^4^, ^5^ In PD, there is failure of the fusion of dorsal and ventral pancreatic buds, which typically should occur at the 6th–8th week of gestation. The duct of Wirsung (duct of the ventral bud) drains only the head of the pancreas via the major papilla, while the duct of Santorini (duct of the dorsal bud) drains the majority of the pancreas, namely body and tail via the more cranially and anteriorly positioned minor papilla.^6^

Although most patients with PD are asymptomatic, PD may be the cause of chronic abdominal pain, acute, and recurrent pancreatitis. The major dominant dorsal pancreatic duct drains into the relatively smaller or stenotic minor papilla, which causes inadequate drainage of pancreatic secretions and transient obstruction of flow. This cause an increase in intraductal pressure and distention of the dorsal duct, which may lead to abdominal pain and pancreatitis.^7^

Herein, we report a case that presented to us with epigastric pain and vomiting, and imagings were consistent with PD. This case report is presented with the aim of highlighting the radiological finding in the PD as well as reminding the reader that PD is one the causes of unexplained abdominal pain, as an early radiological diagnosis can prompt early surgical intervention if necessary.

## 2 CASE PRESENTATION

A 16‐year‐old man presented to the emergency department with dull aching epigastric nonradiating pain for a day, which was increasing in severity. He also had two episodes of nonbilious vomiting and loose stool without fever. He had a past history of similar epigastric pain requiring inpatient care 4 years ago that responded to conservative management. The report of the computed tomography (CT) scan done at that time mentioned mesenteric lymphadenopathy only. He had been evaluated for similar pain of lesser magnitude many times elsewhere and was treated intermittently with oral proton pump inhibitors and analgesics. The patient gave no history of smoking or drinking.

**FIGURE 1:**
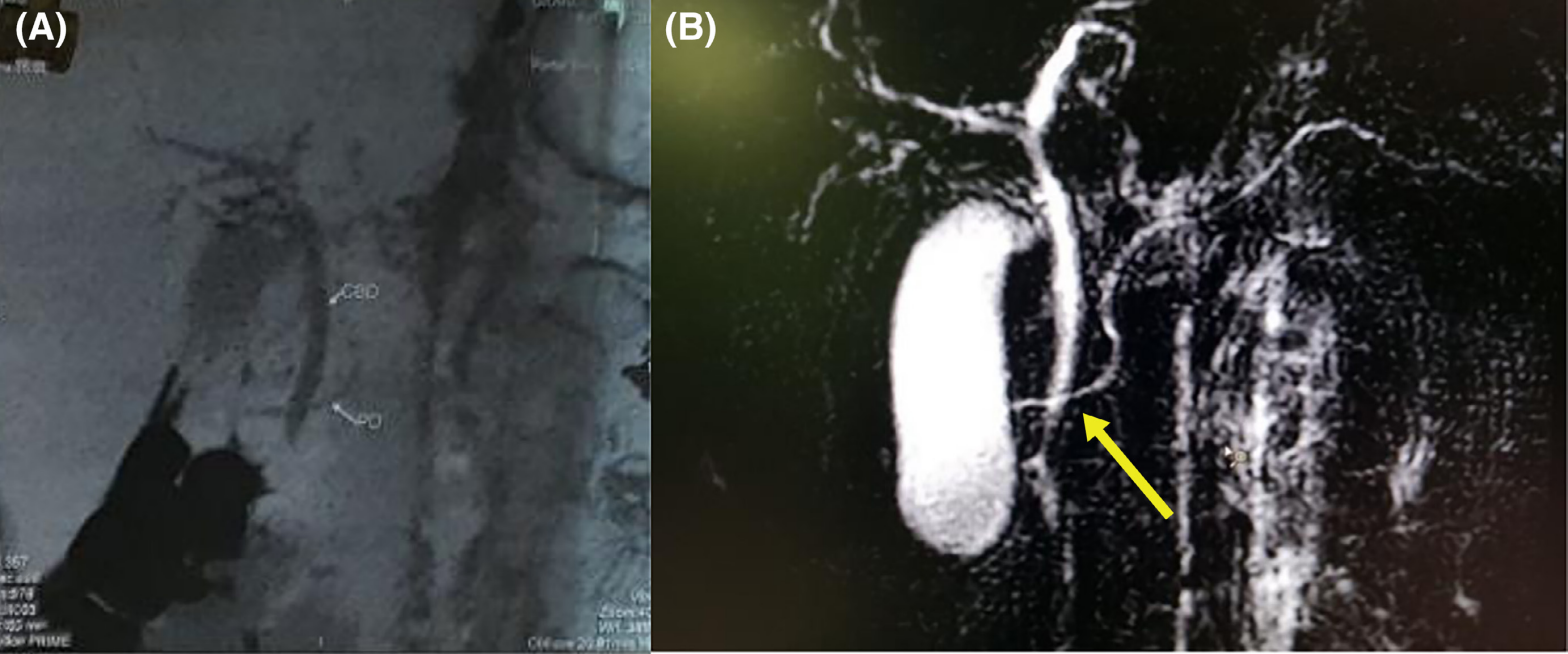
(A) CECT thick slab coronal view. (B) Thick‐slab coronal MRCP heavily T2‐weighted sequence, showing the dominant dorsal duct crossing the intrapancreatic common bile duct giving crossing duct sign. The CBD courses toward the major papilla. The dorsal pancreatic duct drains separately into the minor papilla. Ventral pancreatic duct is not visualized.

On examination, his vitals were normal. No pallor or icterus were noted, and the patient was not dehydrated. An abdominal examination showed mild tenderness over the epigastric region. His laboratory reports showed normal liver function tests and pancreatic enzymes. A complete blood count was normal. The ultrasonography examination was normal. Based on clinical symptomatology and tests, he was managed in the line of acute gastroenteritis and was given antacids, analgesics, antispasmodics, and intravenous fluids. During the course of treatment, he developed increasingly severe pain, requiring stronger analgesics (opioids) with increasing frequency. In view of increasing pain, contrast‐enhanced CT (CECT) of the abdomen was done, which was suggestive of a bulky pancreas. The dominant dorsal pancreatic duct was draining into the minor papillae rather than the major papillae, suggesting pancreas divisum, and the main pancreatic duct was crossing the intrapancreatic common bile duct, which was further confirmed with MRCP as shown in Figure 1. The patient was counseled and referred to surgical gastroenterology for further management.

## 3 DISCUSSION

Pancreas divisum has three major types. Type I, or classic PD, is where there is a complete failure of the dorsal and ventral buds to fuse. In type II PD, there is no ventral duct as in our case; hence, the minor papilla drains the entire pancreas and the major papilla drains some of the common bile duct. Finally, type III has a small remnant of communication between the dorsal duct and ventral duct.^8^

Pancreas divisum, though mostly asymptomatic, may present as the etiology of acute or recurrent pancreatitis, chronic pancreatitis, or chronic abdominal pain.^9^ The revised Atlanta classification requires that two or more of the following criteria be met for the diagnosis of acute pancreatitis: (a) abdominal pain suggestive of pancreatitis, (b) serum amylase or lipase level greater than three times the upper normal value, or (c) characteristic imaging findings.^10^ Chronic abdominal pain can be defined as a pain syndrome consistent with pancreatitis, but an identifiable etiological cause cannot be established.^9^ Usually, pancreatic enzymes are not elevated, and imaging studies do not reveal any abnormalities. Our patient had epigastric pain, but pancreatic enzymes were normal. Although the pancreas was bulky, no peripancreatic fat stranding or collection was noted for definite evidence of pancreatic inflammation.

Endoscopic retrograde cholangiopancreatography is the modality of choice for diagnosing PD; however, the effects related to it—a likely invasive test requiring sedation, 10%–15% complication rate, and up to 10% post‐ERCP pancreatitis—cannot be overlooked.^11^, ^12^, ^13^ In fact, high‐resolution T2‐weighted images in MRCP can also well‐visualize the ducts due to the signal from the fluid content in the duct. Magnetic resonance cholangiopancreatography is a noninvasive diagnostic technique to visualize the biliary tree and the pancreatic duct. It has similar accuracy as compared to ERCP for the diagnosis of PD.^14^, ^15^

The predominant pancreatic duct drains into the minor papilla, which occurs at a level superior to the level of the bile duct opening. The bile duct drains into the minor papilla. This appearance of the dorsal duct running across the intrapancreatic common bile duct can be depicted as a crossing duct sign. This indicates PD, which can be seen on MRCP images. Sometimes, the duct of the ventral system may not be visible due to its small caliber. A focal dilation of the terminal duct of Santorini (dorsal duct) can be seen and is called Santorinicele.^14^, ^16^

Similarly, with the advent of multidetector CT scanners and high‐spatial‐resolution thin‐section imaging, PD may be routinely seen with the use of CT as well.^17^ The CECT of this patient also showed that the dorsal duct was prominent and draining into the minor papilla, suggestive of PD.

For patients with abdominal pain as a result of PD, there is a corrective surgical option available. The patient may elect to receive endoscopic stenting or sphincterotomy of a minor papilla.^11^ Detailed radiological imaging can pick up these correctable congenital defects, hence necessitating an early radiological diagnosis.

## 4 CONCLUSION

In this case report, we attempted to highlight the radiological findings in the patient with PD. The patient did not have biochemical and radiological evidence of pancreatitis, but this is surgically correctable in symptomatic patients; hence, early radiological detection is important. Looking for the crossing duct sign in the coronal thick slab images in CT as well as MRCP is sufficient to diagnose PD.

## AUTHOR CONTRIBUTIONS

**Prakash Kayastha:** Conceptualization; supervision; writing – original draft; writing – review and editing. **Biraj Pokhrel:** Resources; supervision; writing – original draft; writing – review and editing. **Uma Bhatta:** Conceptualization; writing – original draft; writing – review and editing. **Roshan Pathak:** Conceptualization; resources; writing – original draft; writing – review and editing. **Goody Jha:** Writing – original draft. **Sharma Paudel:** Supervision; writing – review and editing. **sundar suwal:** Supervision. **Dosti Regmi:** Conceptualization; writing – original draft; writing – review and editing.

## CONFLICT OF INTEREST STATEMENT

The authors declare that there is no conflict of interest regarding the publication of this paper.

## ETHICS STATEMENT

Need for ethics approval waived. Consent from the patient deemed to be enough.

## CONSENT

Written informed consent was obtained from the patient for publication of this case report and any accompanying images. A copy of the written consent will be available for review by the editor‐in‐chief of this journal if requested.

## Data Availability

Not applicable.
